# Late Presentation of Pulmonary Aplasia in Adulthood: A Report of a Rare Case

**DOI:** 10.7759/cureus.62647

**Published:** 2024-06-18

**Authors:** Lokesh Devalla, Babaji Ghewade, Ulhas Jadhav, Pankaj Wagh, Vivek D Alone

**Affiliations:** 1 Respiratory Medicine, Jawaharlal Nehru Medical College, Datta Meghe Institute of Medical Sciences, Wardha, IND

**Keywords:** cough, breathlessnes, pulmonary rehabilitation and medicine, pulmonary angiogram, opaque hemithorax

## Abstract

Aplasia of the lung is an uncommon congenital anomaly that can resemble several common illnesses radiologically and presents as an opaque hemithorax with ipsilateral displacement of the mediastinum. This case scenario involves a young woman who has been experiencing recurrent pulmonary tract infections and worsening dyspnea since childhood, presenting as pneumonic consolidation on a chest X-ray. The case explores the importance of lung scans, CT pulmonary angiography, and bronchoscopy to elicit the absence of lung parenchyma on one side.

## Introduction

Three conditions together make up the uncommon lung disorder known as unilateral pulmonary underdevelopment: pulmonary hypoplasia, pulmonary aplasia, and pulmonary agenesis. The absence of the lung parenchyma and its arteries with a blind end bronchial stump is known as lung aplasia. Since Morgagni's initial report in 1762, about 200 cases have been described in the literature [1]. Lung agenesis and aplasia had a cumulative incidence of 0.0034-0.0097%. When a patient has a fully opaque hemithorax, it should be considered in conjunction with other hereditary abnormalities. Because this illness is rare, there isn't much information available about its clinical trajectory [2]. Bilateral aplasia is very rare and is not life-compatible [3].

## Case presentation

A 19-year-old female patient came to the hospital with complaints of an eight-day cold, dry cough, and exertional dyspnea with a pulse rate of 87 beats per minute, respiratory rate of 21 per minute, saturation of 95% on room air, and blood pressure of 100/70 mm of Hg. She denied having ever experienced palpitations, hemoptysis, fever, chest pain, or weight loss. She had a substantial prior medical history of recurrent upper respiratory tract infections that began when she was 10 years old and for which she always received symptomatic treatment. She was appropriately inoculated and her birth history was uncomplicated. Her general examination revealed nothing unusual. Examination of the respiratory system indicated a non-localized apex beat, a displaced trachea to the right, and a flattened right hemithorax with reduced motility on the same side. In the right hemithorax, there was a dull percussion note and no breath sounds, except in the right infraclavicular region and the right parasternal line. Auscultation revealed no added sounds. The other systems were clinically normal. A routine blood test revealed normal biochemistry and a full hemogram.

**Figure 1:**
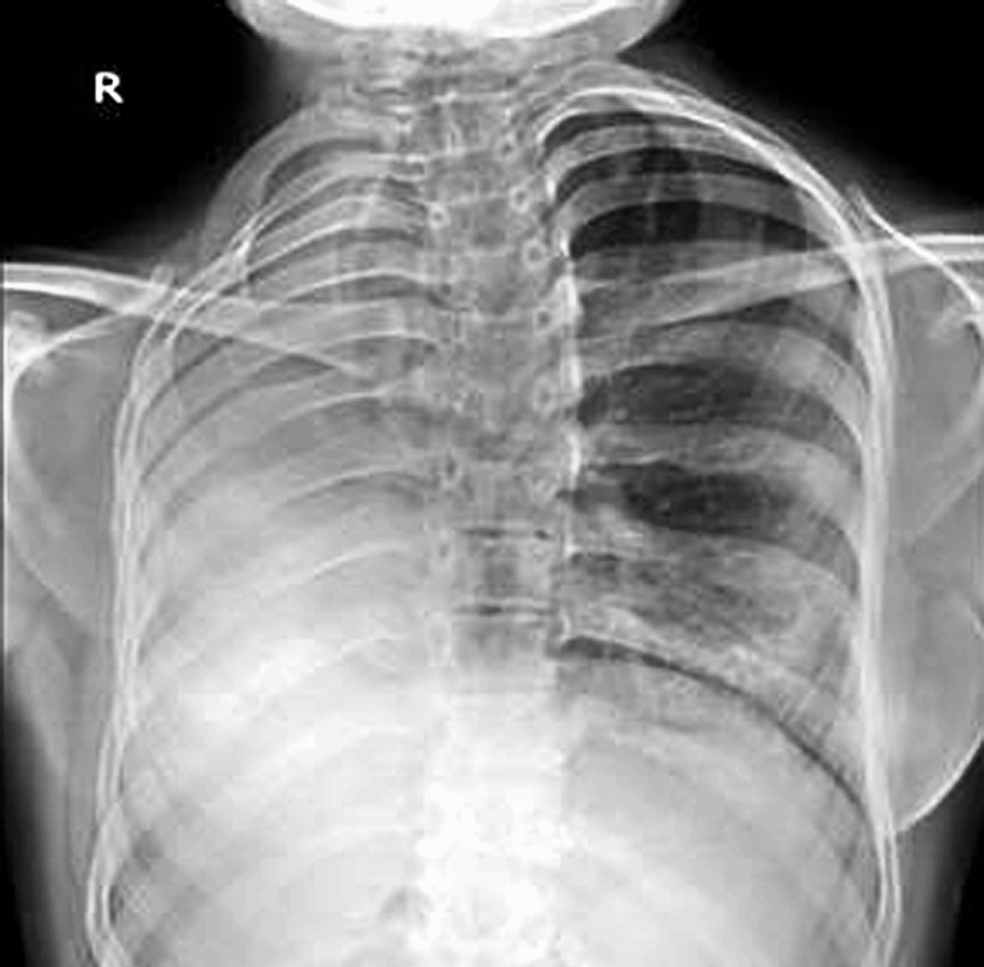
Chest X-ray posteroanterior (PA) view revealing a dense radiopaque shadow in the right lower hemithorax with ipsilateral mediastinal shift, rib crowding, and elevation of right hemidiaphragm

She was negative for sputum acid-fast bacilli and culture. Her chest radiograph showed an opaque homogeneous opacity in the right hemithorax with rib crowding and same-sided mediastinal shift (Figure 1).

**Figure 2:**
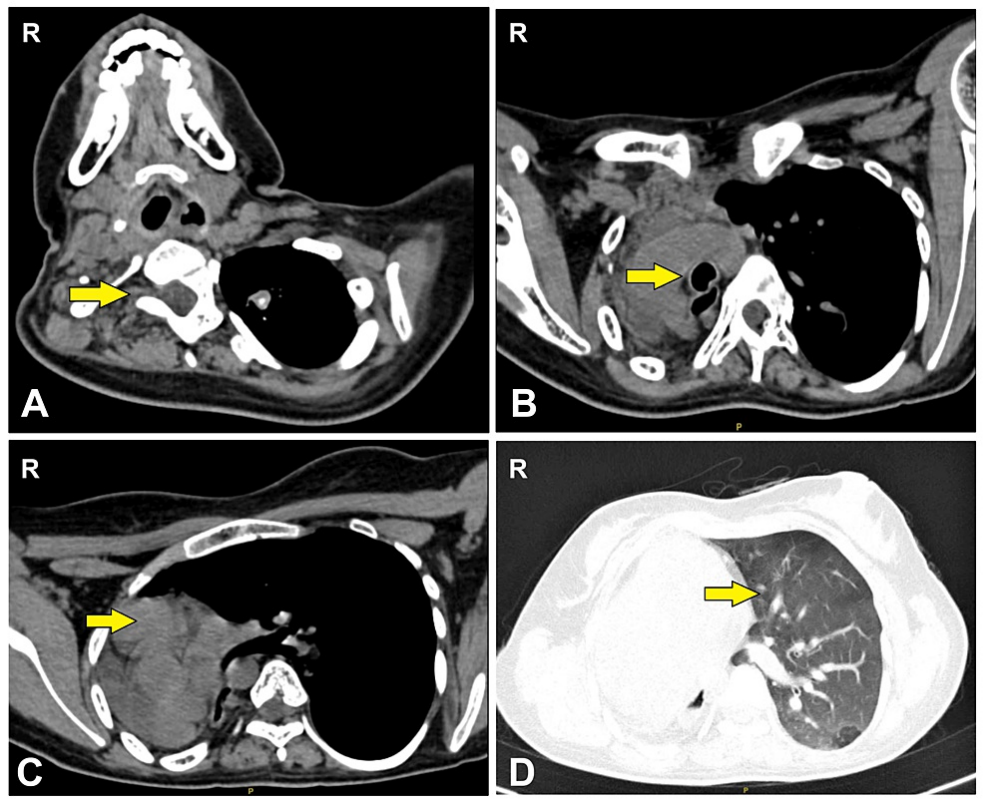
High-resolution computed tomography (HRCT) thorax showing (A) absence of right lung parenchyma, (B) rightward mediastinal shift of bronchial tree, (C) dextrocardia, and (D) normal left lung parenchyma (indicated by yellow arrows)

High-resolution computed tomography (HRCT) thorax showed a lack of right lung parenchyma with left lung herniation into the right hemithorax pushing the heart right (dextrocardia) and posteriorly with rightward mediastinal shift (Figure 2).

**Figure 3:**
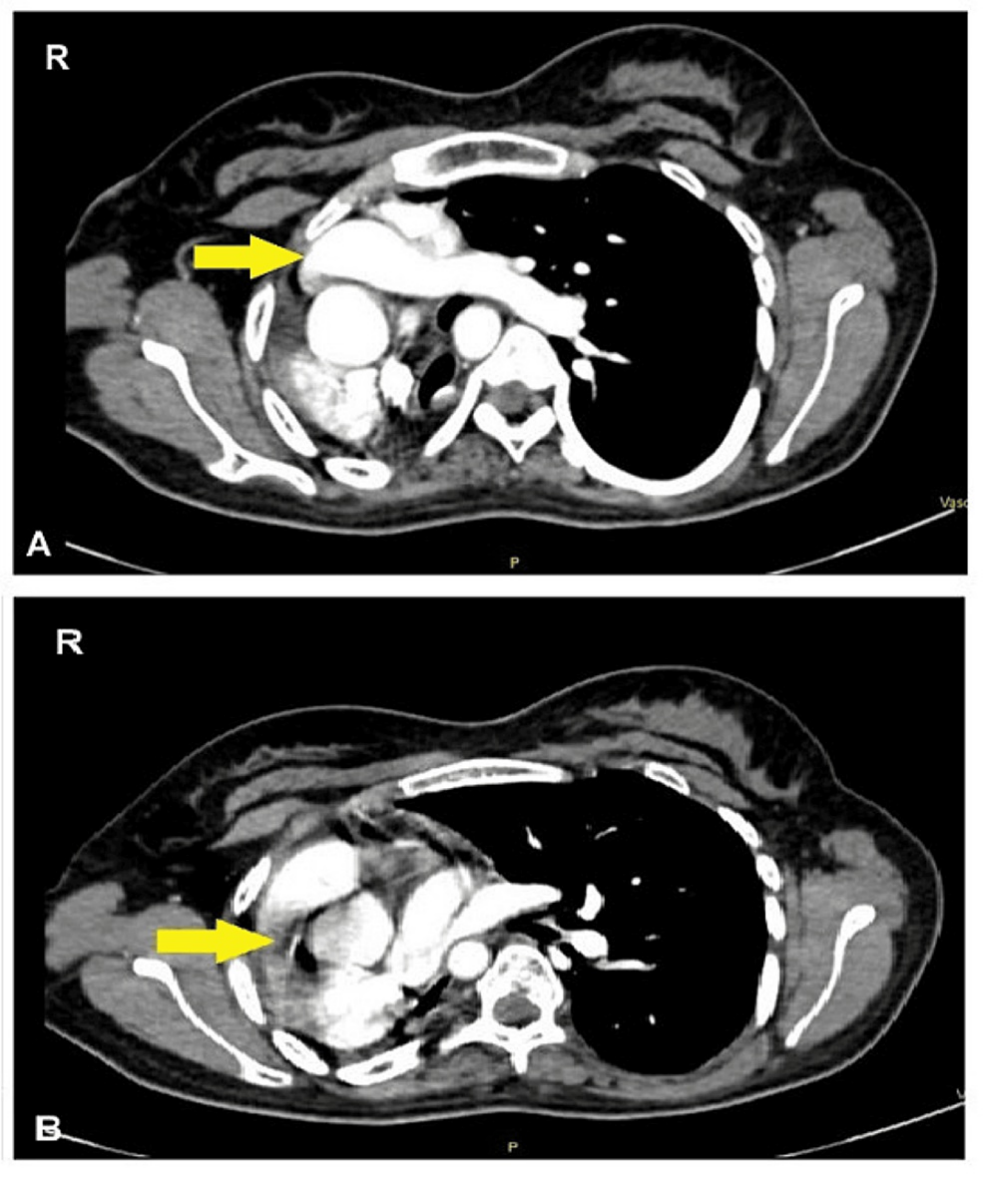
CT pulmonary angiogram suggesting (A) evidence of left pulmonary artery with (B) absence of right pulmonary artery (indicated by yellow arrows)

Based on seemingly interesting HRCT findings, CT pulmonary angiography was done that revealed the presence of the left pulmonary artery from its origin and non-visualization of the right pulmonary artery along with right lung parenchyma and right-sided mediastinal shift of left lung tissue and severe dextro position of the heart (Figure 3).

**Figure 4:**
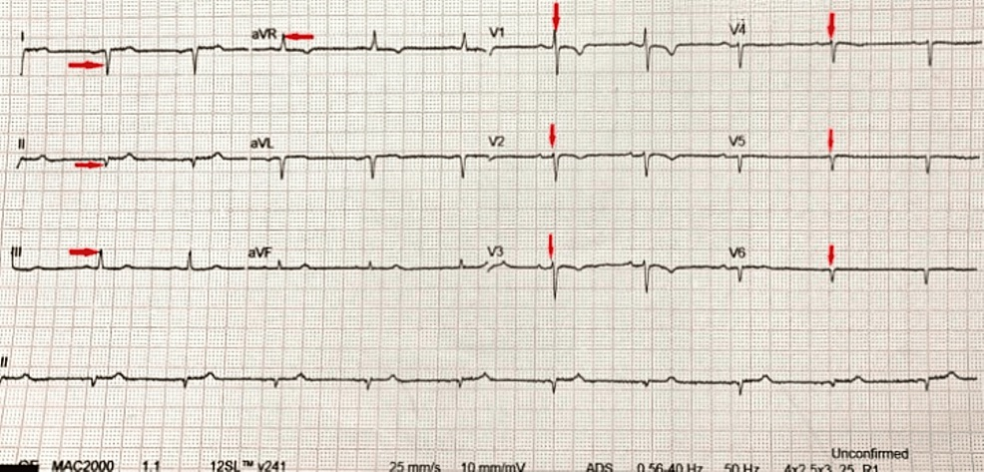
ECG revealing right axis deviation (RAD), positive QRS in lead aVR, inverted QRS in lead I, and absent R wave progression suggestive of dextrocardia

**Figure 5:**
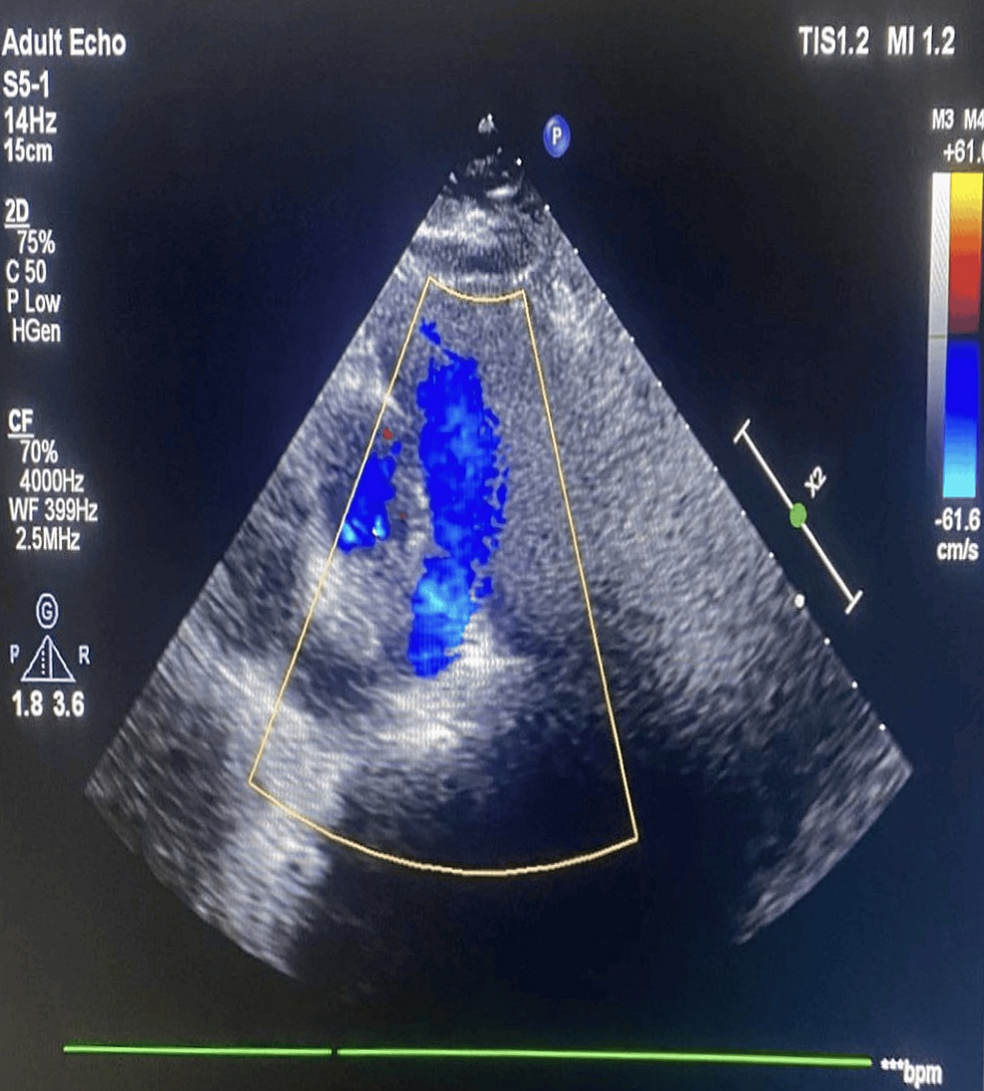
2D echocardiography (ECHO) showing absent right pulmonary artery

Abdomen and pelvic ultrasonography did not show any abnormalities. ECG changes revealed a dextro-posed heart (Figure 4), while trans-thoracic echocardiography showed a structurally normal heart with no pulmonary arterial hypertension (PAH) and valvular defects with an absence of the right pulmonary artery (Figure 5).

In order to assess the lung functioning capacity, the patient was made to execute a pulmonary function test (PFT), which showed a reduction of forced expiratory volume in the first second (FEV1) of less than 80% with a ratio of FEV1/forced vital capacity (FVC) greater than equal to 70%, thus suggesting a restrictive pattern of pulmonary disease (Table 1).

**Table 1:** Spirometry (PFT) showing restrictive lung pattern FVC: Forced vital capacity (FVC); FEV1: Forced expiratory volume in the first second; FEF25-75: Forced expiratory flow at 25-75% of FVC; PEFR: Peak expiratory flow rate (PEFR); PFT: Pulmonary function test

Parameter	Predicted	Pre-bronchodilator	% Predicted pre-dilator	Post-bronchodilator	% Predicted post-dilator	Improvement
FVC (L)	3.43	1.42	41	1.44	42	01
FEV1 (L)	2.80	1.22	43	1.30	46	03
FEV1/FVC (%)	0.81	0.85	104	0.90	111	07
FEF25-75 (L/S)	3.23	1.93	60	2.06	64	04
PEFR (L/S)	7.85	4.24	54	4.42	56	02

**Figure 6:**
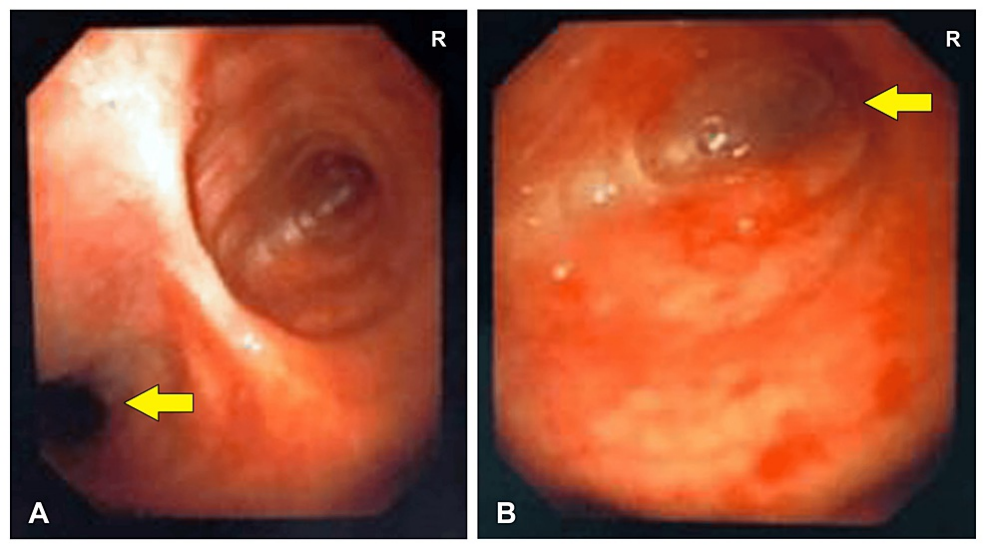
Fibre-optic bronchoscopy showing (A) a normal left main bronchus with (B) a blind ending of the rudimentary right main bronchus (indicated by yellow arrows)

Further out of curiosity and following the fiber-optic bronchoscopy procedure, the patient's right main bronchus had a rudimentary blind ending within two centimeters of the carina, but the left side bronchial tree appeared normal without any signs of infection (Figure 6).

Following a thorough investigative workup, right pulmonary aplasia was ultimately diagnosed. After receiving oral antibiotics, expectorants, and bronchodilators for five days, the patient's symptoms improved. She was then discharged from the hospital and advised vaccinations with regular follow-ups.

## Discussion

Unilateral lung aplasia or agenesis may occur in different degrees of severity. Males predominate over females, the affected lung is often the left than the right, and most cases show other congenital abnormalities such as cardiac malformation, pulmonary artery atresia, tracheoesophageal fistula, horseshoe kidney, etc. However, some previous reports show that other anomalies are more commonly associated with right-sided aplasia, and people with right-sided aplasia typically die within the first year of life due to related cardiac deformities.

Originally, Schneider 1912 classified agenesis into three groups explained by Booth et al. [4], which were later on modified by Boyden [5]. Type I (Agenesis): Complete absence of lung and bronchus and absence of blood vessels to the affected side. Type II (Aplasia): Rudimentary bronchus without lung tissue. Type III (Hypoplasia): Presence of variable amounts of the lung parenchyma, bronchial tree, and supporting vasculature. Schneider's agenesis grades I and II are distinguished by the absence of lung tissue on the affected side while a branch from the main pulmonary artery vascularizes the other side.

A possible nidus for contaminated secretions is this rudimentary bronchus, and the secretions may go to the opposite airway and cause recurrent pneumonia [6]. Most patients exhibit symptoms during infancy such as recurrent cough with or without expectoration, dyspnea, fever, and weight loss [1]. Adults, on the other hand, are typically symptom-free [2]. Although some patients may experience repeated lung infections from bronchitis or bronchiectasis, HRCT thorax had no evident lesions in the left lung of this patient. On the chest radiograph, the case appeared as a tiny, dense hemithorax with over-distension and herniation of the opposite lung, making it difficult to distinguish from collapse or other causes of endobronchial blockage. HRCT thorax can detect the absence of lung parenchyma and pulmonary artery with a bronchial stump on the affected side [7]. Thus, CT is a more trustworthy modality for examining congenital lung abnormalities and should be employed before more invasive procedures like bronchography or aortography [8]. Bronchoscopy helped in revealing the main bronchus, which terminated in a blind-ending pouch.

Management includes vaccination, pulmonary rehabilitation (to improve bronchial cleanliness), oxygen assistance (if hypoxemic), and antibiotic therapy for infections. The literature indicates that there is not a particular treatment. We administered antibiotics, and supportive care, assessed the patient for pulmonary hypertension, and recommended routine vaccination and chest physiotherapy follow-up. There are not many examples of aortopexy or diaphragmatic translocation effectively handled in the literature. Techniques such as reducing heart rotation, altering mediastinal position, relieving tracheal kinks, and inflation of lung parenchyma [9] can aid in patient recovery from respiratory distress. However, there are several risks associated with any surgical intervention, so before planning a treatment, the patient needs to be carefully assessed.

## Conclusions

Clinically, pulmonary aplasia is a rare condition. When a radiopaque hemithorax is present, there needs to be a high index of suspicion because clinical presentation varies greatly. Chest X-rays, HRCT thorax, and lung scans are helpful diagnostic techniques. Regarding pulmonary vasculature, further information can be obtained with a CT pulmonary angiogram and two-dimensional echocardiogram. A bronchoscopy also helps to confirm the diagnosis. Management is mainly symptomatic and supportive.
